# 3,5-Bis[(pyridin-4-yl)meth­oxy]benzoic acid

**DOI:** 10.1107/S1600536812049550

**Published:** 2012-12-12

**Authors:** Hong Lin, Yi-Ping Zhang

**Affiliations:** a Jinhua Professional Technical College, No. 1188 Wuzhou Street, Jinhua, Zhejiang 321017, People’s Republic of China; b Zhejiang Key Laboratory for Reactive Chemistry on Solid Surfaces, Institute of Physical Chemistry, Zhejiang Normal University, Jinhua, Zhejiang 321004, People’s Republic of China

## Abstract

Single crystals of the title compound, C_19_H_16_N_2_O_4_, were obtained under hydro­thermal conditions by an unintended recrystallization of the employed microcrystalline starting material. The [(pyridin-4-yl)meth­oxy]benzoic acid unit is nearly planar, with a maximum deviation from the least-squares plane of 0.194 (2) Å. This plane is inclined by 35.82 (6)° to that defined by the second (pyridin-4-yl)meth­oxy group [in which the largest deviation from the least-squares plane is 0.013 (2) Å]. In the crystal, mol­ecules are linked by O—H⋯N hydrogen bonds involving the acid hy­droxy group and a pyridine N atom into chains parallel to [-201].

## Related literature

For compounds with metal-organic framework structures derived from the title compound, see: Xu *et al.* (2009 ▶).
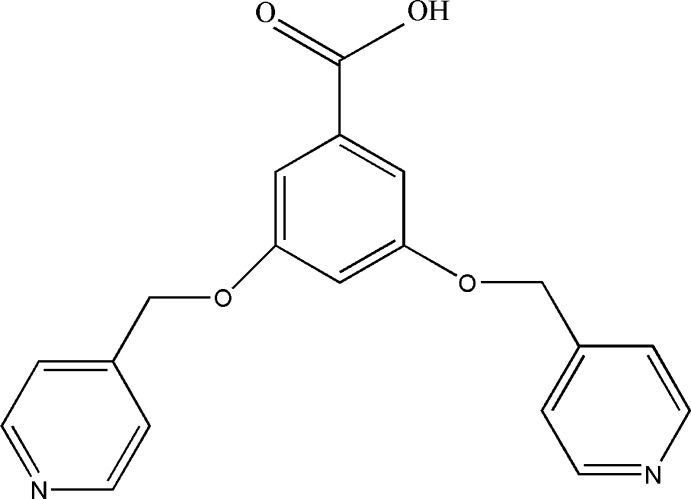


## Experimental

### 

#### Crystal data

C_19_H_16_N_2_O_4_
*M* *_r_* = 336.34
Monoclinic, 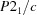

*a* = 11.1523 (6) Å
*b* = 11.2120 (6) Å
*c* = 13.9255 (7) Å
β = 102.827 (3)°
*V* = 1697.79 (15) Å^3^
*Z* = 4
Mo *K*α radiation
μ = 0.09 mm^−1^
*T* = 296 K
0.38 × 0.33 × 0.21 mm

#### Data collection

Bruker SMART CCD diffractometer
Absorption correction: multi-scan (*SADABS*; Bruker, 2006 ▶) *T*_min_ = 0.965, *T*_max_ = 0.980
25948 measured reflections
3936 independent reflections
2980 reflections with *I* > 2σ(*I*)
*R*_int_ = 0.025

#### Refinement

*R*[*F* ^2^ > 2σ(*F* ^2^)] = 0.046
*wR*(*F* ^2^) = 0.132
*S* = 1.04
3936 reflections
226 parameters
1 restraint
H-atom parameters constrained
Δρ_max_ = 0.26 e Å^−3^
Δρ_min_ = −0.25 e Å^−3^

### 

Data collection: *SMART* (Bruker, 2006 ▶); cell refinement: *SAINT* (Bruker, 2006 ▶); data reduction: *SAINT*; program(s) used to solve structure: *SHELXS97* (Sheldrick, 2008 ▶); program(s) used to refine structure: *SHELXL97* (Sheldrick, 2008 ▶); molecular graphics: *Mercury* (Macrae *et al.*, 2006 ▶); software used to prepare material for publication: *publCIF* (Westrip, 2010 ▶).

## Supplementary Material

Crystal structure: contains datablock(s) I, global. DOI: 10.1107/S1600536812049550/wm2702sup1.cif

Structure factors: contains datablock(s) I. DOI: 10.1107/S1600536812049550/wm2702Isup2.hkl

Supplementary material file. DOI: 10.1107/S1600536812049550/wm2702Isup3.cml

Additional supplementary materials:  crystallographic information; 3D view; checkCIF report

## Figures and Tables

**Table 1:** Hydrogen-bond geometry (Å, °)

*D*—H⋯*A*	*D*—H	H⋯*A*	*D*⋯*A*	*D*—H⋯*A*
O2—H1*A*⋯N2^i^	0.85	1.83	2.6736 (16)	171

Symmetry code: (i) 
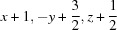
.

**Fig. 1.**
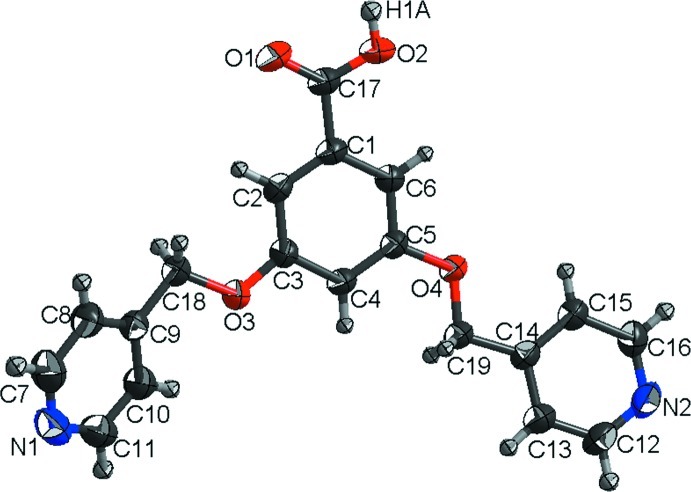
The molecular structure of (I), showing the atom-numbering scheme. Displacement ellipsoids are drawn at the 30% probability level.

**Fig. 2.**
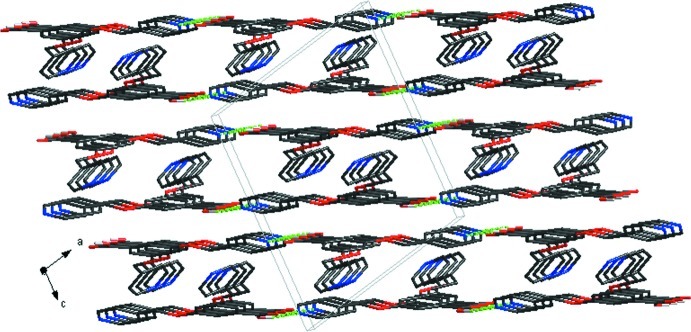
A view of the one-dimensional chain-like structure of (I).

## supplementary crystallographic information

### Comment

MOFs derived from the 3,5-bis(pyridin-4-ylmethoxy)benzoic acid ligand have been synthesized recently (Xu *et al.*, 2009). During hydrothermal syntheses intended for crystal growth of related systems, crystals of the educt 3,5-bis(pyridin-4-yl-methoxy)benzoic acid, (I), have been unexpectedly obtained.

The molecular structure of (I) is presented in Fig. 1. Atoms O1—O4, C1—C6, C12—C19 and N2 are nearly coplanar (r.m.s. deviation = 0.098 Å; largest deviation from the least-squares plane 0.194 (2) Å); atoms C7—C11, C18 and N1 of the second pyridyl moiety are in another plane (r.m.s. deviation = 0.003 Å; largest deviation from the least-squares plane 0.013 (2) Å). The dihedral angle between the two planes is 35.82 (6)°.

The carboxylic O—H group and neighbouring pyridyl N atoms are involved in O—H···N hydrogen-bonding interactions (Table 1), forming chains extending parallel to [201] (Fig. 2). There are no significant π···π interactions between the aromatic planes of adjacent chains.

### Experimental

3,5-bis(pyridin-4-yl-methoxy)benzoic acid was obtained commercially. A mixture of 3,5-bis(pyridin-4-yl-methoxy)benzoic acid (0.5 mmol), CdCl_2_^.^2.5H_2_O (0.25 mmol), and NaOH (0.5 mmol) in H_2_O (16 ml) was sealed in a 25 ml stainless steel reactor with a telflon liner and heated at 433 K for 72 h, and then cooled to room temperature at a speed of 5 Kh^-1^. Colourless single crystals of (I) were obtained by slow evaporation of the filtrate over a few days.

### Refinement

Carbon-bound H-atoms were positioned geometrically and included in the refinement using a riding model [C—H 0.93 Å (aromatic), 0.97 Å (methylene); *U*_iso_(H) = 1.2*U*_eq_(C)]. The oxygen-bound H-atom was located in a difference Fourier map and refined with an O—H distance restrained to 0.85 Å [*U*_iso_(H) = 1.2*U*_eq_(O)].

### Crystal data

**Table:**

C_19_H_16_N_2_O_4_	*F*(000) = 704
*M**_r_* = 336.34	*D*_x_ = 1.316 Mg m^−^^3^
Monoclinic, *P*2_1_/*c*	Mo *K*α radiation, λ = 0.71073 Å
Hall symbol: -P 2ybc	Cell parameters from 9997 reflections
*a* = 11.1523 (6) Å	θ = 1.9–27.6°
*b* = 11.2120 (6) Å	µ = 0.09 mm^−^^1^
*c* = 13.9255 (7) Å	*T* = 296 K
β = 102.827 (3)°	Block, colourless
*V* = 1697.79 (15) Å^3^	0.38 × 0.33 × 0.21 mm
*Z* = 4	

### Data collection

**Table:**

Bruker SMART CCD diffractometer	3936 independent reflections
Radiation source: fine-focus sealed tube	2980 reflections with *I* > 2σ(*I*)
Graphite monochromator	*R*_int_ = 0.025
ω scans	θ_max_ = 27.6°, θ_min_ = 1.9°
Absorption correction: multi-scan (*SADABS*; Bruker, 2006)	*h* = −13→14
*T*_min_ = 0.965, *T*_max_ = 0.980	*k* = −14→14
25948 measured reflections	*l* = −18→18

### Refinement

**Table:**

Refinement on *F*^2^	Primary atom site location: structure-invariant direct methods
Least-squares matrix: full	Secondary atom site location: difference Fourier map
*R*[*F*^2^ > 2σ(*F*^2^)] = 0.046	Hydrogen site location: inferred from neighbouring sites
*wR*(*F*^2^) = 0.132	H-atom parameters constrained
*S* = 1.04	*w* = 1/[σ^2^(*F*_o_^2^) + (0.0639*P*)^2^ + 0.2876*P*] where *P* = (*F*_o_^2^ + 2*F*_c_^2^)/3
3936 reflections	(Δ/σ)_max_ = 0.001
226 parameters	Δρ_max_ = 0.26 e Å^−^^3^
1 restraint	Δρ_min_ = −0.25 e Å^−^^3^

### Special details

**Table:**

Geometry. All e.s.d.'s (except the e.s.d. in the dihedral angle between two l.s. planes) are estimated using the full covariance matrix. The cell e.s.d.'s are taken into account individually in the estimation of e.s.d.'s in distances, angles and torsion angles; correlations between e.s.d.'s in cell parameters are only used when they are defined by crystal symmetry. An approximate (isotropic) treatment of cell e.s.d.'s is used for estimating e.s.d.'s involving l.s. planes.
Refinement. Refinement of *F*^2^ against ALL reflections. The weighted *R*-factor *wR* and goodness of fit *S* are based on *F*^2^, conventional *R*-factors *R* are based on *F*, with *F* set to zero for negative *F*^2^. The threshold expression of *F*^2^ > σ(*F*^2^) is used only for calculating *R*-factors(gt) *etc*. and is not relevant to the choice of reflections for refinement. *R*-factors based on *F*^2^ are statistically about twice as large as those based on *F*, and *R*- factors based on ALL data will be even larger.

### Fractional atomic coordinates and isotropic or equivalent isotropic displacement parameters (Å^2^)

**Table:**

	*x*	*y*	*z*	*U*_iso_*/*U*_eq_	
N1	0.68638 (16)	−0.22020 (13)	0.06179 (12)	0.0777 (4)	
N2	−0.01294 (12)	0.70984 (13)	−0.08773 (10)	0.0647 (4)	
O1	0.89003 (10)	0.51053 (12)	0.36115 (10)	0.0864 (4)	
O2	0.78235 (10)	0.67629 (10)	0.32201 (9)	0.0756 (4)	
H1A	0.8458	0.7098	0.3565	0.091*	
O3	0.61952 (9)	0.19740 (9)	0.14886 (9)	0.0684 (3)	
O4	0.38875 (8)	0.55619 (8)	0.10585 (7)	0.0537 (3)	
C1	0.69258 (11)	0.49686 (12)	0.25395 (9)	0.0448 (3)	
C2	0.70848 (11)	0.37573 (12)	0.23705 (10)	0.0482 (3)	
H2A	0.7807	0.3368	0.2672	0.058*	
C3	0.61508 (12)	0.31505 (12)	0.17484 (10)	0.0495 (3)	
C4	0.50609 (12)	0.37228 (12)	0.13026 (10)	0.0491 (3)	
H4A	0.4432	0.3302	0.0890	0.059*	
C5	0.49235 (11)	0.49207 (12)	0.14796 (9)	0.0441 (3)	
C6	0.58568 (11)	0.55589 (12)	0.21024 (9)	0.0447 (3)	
H6A	0.5761	0.6366	0.2221	0.054*	
C7	0.79339 (18)	−0.16440 (16)	0.08353 (15)	0.0779 (5)	
H7A	0.8616	−0.2048	0.0720	0.093*	
C8	0.81162 (14)	−0.05037 (14)	0.12215 (12)	0.0618 (4)	
H8A	0.8892	−0.0155	0.1348	0.074*	
C9	0.71223 (12)	0.00998 (12)	0.14135 (9)	0.0473 (3)	
C10	0.59962 (14)	−0.04711 (14)	0.11926 (11)	0.0578 (4)	
H10A	0.5298	−0.0094	0.1307	0.069*	
C11	0.59181 (16)	−0.16036 (15)	0.08012 (13)	0.0687 (4)	
H11A	0.5151	−0.1971	0.0657	0.082*	
C12	−0.00017 (14)	0.59958 (16)	−0.11826 (12)	0.0647 (4)	
H12A	−0.0621	0.5683	−0.1678	0.078*	
C13	0.09988 (13)	0.52908 (15)	−0.08048 (10)	0.0558 (4)	
H13A	0.1048	0.4520	−0.1040	0.067*	
C14	0.19354 (11)	0.57435 (12)	−0.00677 (9)	0.0445 (3)	
C15	0.18240 (13)	0.68999 (13)	0.02401 (10)	0.0511 (3)	
H15A	0.2440	0.7243	0.0722	0.061*	
C16	0.07743 (14)	0.75435 (14)	−0.01820 (12)	0.0612 (4)	
H16A	0.0700	0.8321	0.0031	0.073*	
C17	0.79843 (12)	0.56116 (13)	0.31786 (11)	0.0541 (3)	
C18	0.72785 (12)	0.13267 (12)	0.18610 (11)	0.0522 (3)	
H18A	0.7424	0.1273	0.2573	0.063*	
H18B	0.7976	0.1723	0.1691	0.063*	
C19	0.29911 (11)	0.49396 (12)	0.03536 (10)	0.0497 (3)	
H19A	0.2697	0.4258	0.0664	0.060*	
H19B	0.3359	0.4649	−0.0171	0.060*	

### Atomic displacement parameters (Å^2^)

**Table:**

	*U*^11^	*U*^22^	*U*^33^	*U*^12^	*U*^13^	*U*^23^
N1	0.0886 (11)	0.0541 (8)	0.0904 (10)	0.0016 (8)	0.0201 (9)	−0.0160 (7)
N2	0.0505 (7)	0.0672 (8)	0.0686 (8)	0.0100 (6)	−0.0036 (6)	0.0160 (7)
O1	0.0504 (6)	0.0758 (8)	0.1111 (10)	0.0056 (6)	−0.0290 (6)	−0.0171 (7)
O2	0.0566 (6)	0.0573 (7)	0.0939 (8)	−0.0075 (5)	−0.0237 (6)	−0.0146 (6)
O3	0.0498 (6)	0.0426 (5)	0.0982 (8)	0.0087 (4)	−0.0150 (5)	−0.0135 (5)
O4	0.0401 (5)	0.0459 (5)	0.0648 (6)	0.0070 (4)	−0.0104 (4)	−0.0112 (4)
C1	0.0367 (6)	0.0487 (7)	0.0453 (6)	−0.0037 (5)	0.0014 (5)	−0.0028 (5)
C2	0.0371 (6)	0.0481 (7)	0.0538 (7)	0.0041 (5)	−0.0016 (5)	0.0013 (6)
C3	0.0427 (7)	0.0416 (7)	0.0598 (8)	0.0021 (5)	0.0017 (6)	−0.0033 (6)
C4	0.0365 (6)	0.0461 (7)	0.0585 (8)	−0.0002 (5)	−0.0030 (5)	−0.0074 (6)
C5	0.0352 (6)	0.0446 (7)	0.0489 (7)	0.0030 (5)	0.0014 (5)	−0.0028 (5)
C6	0.0415 (7)	0.0406 (7)	0.0484 (7)	−0.0010 (5)	0.0022 (5)	−0.0041 (5)
C7	0.0740 (11)	0.0671 (11)	0.0956 (13)	0.0193 (9)	0.0255 (10)	−0.0133 (9)
C8	0.0506 (8)	0.0611 (9)	0.0731 (10)	0.0059 (7)	0.0121 (7)	−0.0047 (7)
C9	0.0500 (7)	0.0447 (7)	0.0442 (6)	0.0053 (6)	0.0044 (5)	0.0015 (5)
C10	0.0510 (8)	0.0553 (8)	0.0660 (9)	0.0029 (6)	0.0107 (7)	−0.0058 (7)
C11	0.0667 (10)	0.0596 (10)	0.0772 (10)	−0.0100 (8)	0.0107 (8)	−0.0104 (8)
C12	0.0496 (8)	0.0758 (11)	0.0586 (8)	0.0018 (7)	−0.0094 (6)	0.0037 (8)
C13	0.0458 (7)	0.0594 (9)	0.0556 (8)	0.0010 (6)	−0.0027 (6)	−0.0052 (6)
C14	0.0368 (6)	0.0499 (7)	0.0444 (6)	0.0030 (5)	0.0038 (5)	0.0013 (5)
C15	0.0459 (7)	0.0497 (8)	0.0533 (7)	0.0037 (6)	0.0014 (6)	0.0009 (6)
C16	0.0577 (9)	0.0513 (8)	0.0718 (9)	0.0105 (7)	0.0083 (7)	0.0068 (7)
C17	0.0425 (7)	0.0551 (8)	0.0578 (8)	−0.0030 (6)	−0.0037 (6)	−0.0074 (6)
C18	0.0453 (7)	0.0486 (7)	0.0569 (8)	0.0060 (6)	−0.0008 (6)	−0.0031 (6)
C19	0.0370 (7)	0.0485 (7)	0.0571 (7)	0.0037 (5)	−0.0036 (6)	−0.0084 (6)

### Geometric parameters (Å, º)

**Table:**

N1—C7	1.322 (2)	C7—H7A	0.9300
N1—C11	1.322 (2)	C8—C9	1.375 (2)
N2—C12	1.325 (2)	C8—H8A	0.9300
N2—C16	1.330 (2)	C9—C10	1.382 (2)
O1—C17	1.2066 (17)	C9—C18	1.5043 (19)
O2—C17	1.3063 (18)	C10—C11	1.377 (2)
O2—H1A	0.8500	C10—H10A	0.9300
O3—C3	1.3716 (17)	C11—H11A	0.9300
O3—C18	1.4060 (16)	C12—C13	1.373 (2)
O4—C5	1.3765 (14)	C12—H12A	0.9300
O4—C19	1.4192 (14)	C13—C14	1.3879 (18)
C1—C6	1.3812 (17)	C13—H13A	0.9300
C1—C2	1.3964 (19)	C14—C15	1.3798 (19)
C1—C17	1.4962 (17)	C14—C19	1.4953 (17)
C2—C3	1.3776 (18)	C15—C16	1.3897 (19)
C2—H2A	0.9300	C15—H15A	0.9300
C3—C4	1.3926 (18)	C16—H16A	0.9300
C4—C5	1.3802 (19)	C18—H18A	0.9700
C4—H4A	0.9300	C18—H18B	0.9700
C5—C6	1.3952 (17)	C19—H19A	0.9700
C6—H6A	0.9300	C19—H19B	0.9700
C7—C8	1.384 (2)		
			
C7—N1—C11	115.73 (15)	C9—C10—H10A	120.3
C12—N2—C16	117.75 (13)	N1—C11—C10	124.16 (16)
C17—O2—H1A	110.9	N1—C11—H11A	117.9
C3—O3—C18	118.52 (10)	C10—C11—H11A	117.9
C5—O4—C19	115.70 (10)	N2—C12—C13	123.37 (14)
C6—C1—C2	121.42 (11)	N2—C12—H12A	118.3
C6—C1—C17	121.41 (12)	C13—C12—H12A	118.3
C2—C1—C17	117.12 (11)	C12—C13—C14	119.14 (14)
C3—C2—C1	118.66 (12)	C12—C13—H13A	120.4
C3—C2—H2A	120.7	C14—C13—H13A	120.4
C1—C2—H2A	120.7	C15—C14—C13	117.94 (12)
O3—C3—C2	125.05 (12)	C15—C14—C19	124.19 (12)
O3—C3—C4	113.87 (11)	C13—C14—C19	117.87 (12)
C2—C3—C4	121.07 (12)	C14—C15—C16	118.82 (13)
C5—C4—C3	119.29 (12)	C14—C15—H15A	120.6
C5—C4—H4A	120.4	C16—C15—H15A	120.6
C3—C4—H4A	120.4	N2—C16—C15	122.96 (14)
O4—C5—C4	123.23 (11)	N2—C16—H16A	118.5
O4—C5—C6	115.90 (11)	C15—C16—H16A	118.5
C4—C5—C6	120.87 (11)	O1—C17—O2	123.51 (13)
C1—C6—C5	118.69 (12)	O1—C17—C1	122.63 (14)
C1—C6—H6A	120.7	O2—C17—C1	113.86 (12)
C5—C6—H6A	120.7	O3—C18—C9	107.95 (11)
N1—C7—C8	124.98 (16)	O3—C18—H18A	110.1
N1—C7—H7A	117.5	C9—C18—H18A	110.1
C8—C7—H7A	117.5	O3—C18—H18B	110.1
C9—C8—C7	118.36 (15)	C9—C18—H18B	110.1
C9—C8—H8A	120.8	H18A—C18—H18B	108.4
C7—C8—H8A	120.8	O4—C19—C14	110.32 (11)
C8—C9—C10	117.45 (13)	O4—C19—H19A	109.6
C8—C9—C18	120.43 (13)	C14—C19—H19A	109.6
C10—C9—C18	122.12 (12)	O4—C19—H19B	109.6
C11—C10—C9	119.32 (14)	C14—C19—H19B	109.6
C11—C10—H10A	120.3	H19A—C19—H19B	108.1
			
C6—C1—C2—C3	0.4 (2)	C18—C9—C10—C11	−178.76 (14)
C17—C1—C2—C3	−176.99 (13)	C7—N1—C11—C10	0.0 (3)
C18—O3—C3—C2	−1.4 (2)	C9—C10—C11—N1	0.1 (3)
C18—O3—C3—C4	177.06 (13)	C16—N2—C12—C13	1.3 (3)
C1—C2—C3—O3	177.65 (14)	N2—C12—C13—C14	−0.3 (3)
C1—C2—C3—C4	−0.7 (2)	C12—C13—C14—C15	−1.1 (2)
O3—C3—C4—C5	−177.79 (13)	C12—C13—C14—C19	177.87 (14)
C2—C3—C4—C5	0.8 (2)	C13—C14—C15—C16	1.5 (2)
C19—O4—C5—C4	−5.20 (19)	C19—C14—C15—C16	−177.46 (13)
C19—O4—C5—C6	174.18 (11)	C12—N2—C16—C15	−0.9 (2)
C3—C4—C5—O4	178.92 (13)	C14—C15—C16—N2	−0.5 (2)
C3—C4—C5—C6	−0.4 (2)	C6—C1—C17—O1	175.83 (15)
C2—C1—C6—C5	−0.1 (2)	C2—C1—C17—O1	−6.8 (2)
C17—C1—C6—C5	177.20 (12)	C6—C1—C17—O2	−4.3 (2)
O4—C5—C6—C1	−179.31 (11)	C2—C1—C17—O2	173.04 (13)
C4—C5—C6—C1	0.1 (2)	C3—O3—C18—C9	−176.09 (13)
C11—N1—C7—C8	−0.7 (3)	C8—C9—C18—O3	147.29 (14)
N1—C7—C8—C9	1.1 (3)	C10—C9—C18—O3	−33.63 (18)
C7—C8—C9—C10	−0.9 (2)	C5—O4—C19—C14	−178.29 (11)
C7—C8—C9—C18	178.20 (15)	C15—C14—C19—O4	−3.97 (19)
C8—C9—C10—C11	0.3 (2)	C13—C14—C19—O4	177.08 (12)

### Hydrogen-bond geometry (Å, º)

**Table:**

*D*—H···*A*	*D*—H	H···*A*	*D*···*A*	*D*—H···*A*
O2—H1*A*···N2^i^	0.85	1.83	2.6736 (16)	171

Symmetry code: (i) *x*+1, −*y*+3/2, *z*+1/2.
